# Alteration of brain metabolism in lacunar stroke based on ^18^F-FDG-PET/MRI analysis

**DOI:** 10.3389/fneur.2025.1710801

**Published:** 2026-01-13

**Authors:** Rong-Rong Huang, Mou-Xiong Zheng, Jia-Jia Wu, Yun-Ting Xiang, Ling-Ling Li, Jing Jin, Jian-Hui He, Xin Gao, Jie Ma, Xu-Yun Hua, Jian-Guang Xu

**Affiliations:** 1 School of Rehabilitation Science, Shanghai University of Traditional Chinese Medicine, Shanghai, China; 2 Engineering Research Center of Traditional Chinese Medicine Intelligent Rehabilitation, Ministry of Education, Shanghai, China; 3 Department of Traumatology and Orthopedics, Shuguang Hospital Affiliated to Shanghai University of Traditional Chinese Medicine, Shanghai, China; 4 Department of Rehabilitation Medicine, Yueyang Hospital of Integrated Traditional Chinese and Western Medicine, Shanghai University of Traditional Chinese Medicine, Shanghai, China; 5 Universal Medical Imaging Diagnostic Center, Shanghai, China

**Keywords:** ^18^F-fluorodeoxyglucose, lacunar, lacunar stroke, metabolism, PET/MRI, standardized uptake value

## Abstract

**Background and Purpose:**

Lacunar stroke (LS) is caused by occlusion of the penetrating branches of the major cerebral arteries and includes small and deep infarcts. Our study aimed to explore brain metabolic alterations in LS.

**Methods:**

Seventy individuals with LS (aged 53.89 ± 1.198 years, 52 males) and 70 healthy controls (HCs; aged 50.34 ± 0.737 years, 42 males) underwent brain 18F-fluorodeoxyglucose positron emission tomography/magnetic resonance imaging. Glucose uptake, metabolic connectivity, and metabolic networks at the group level were analyzed.

**Results:**

Compared with HCs, LS patients exhibited a higher standardized uptake value in the right postcentral gyrus (*p* < 0.001) and lower metabolic connectivity between the right postcentral gyrus and the right caudate nucleus, left amygdala, left hippocampus, and left supramarginal gyrus (*p* < 0.001). In the analysis of network properties, compared with HCs, LS patients demonstrated higher clustering coefficient (*p* < 0.001), global efficiency (*p* < 0.001), local efficiency (*p* < 0.001), gamma (*p* = 0.024), and lambda (*p* = 0.016), as well as a shorter path length (*p* < 0.001). Additionally, we observed a higher degree in the right superior temporal gyrus (*p* = 0.002), greater efficiency in the middle part of the right superior frontal gyrus (*p* = 0.004), and a lower degree in the left insula (*p* = 0.002).

**Conclusions:**

Our study identified that LS is not merely localized brain damage; it also involves broader dysfunction across brain networks, thereby affecting advanced cognitive functions. The observed compensatory increase in global network efficiency in LS patients might serve to maintain cerebral glucose metabolism. These findings may indicate new target areas for future treatments.

## 1 Introduction

Lacunar stroke (LS) accounts for roughly 25% of ischemic strokes (1, 2). It is primarily caused by the occlusion of small penetrating arteries that arise from the circle of Willis or its proximal branches, and is commonly associated with sensory and motor impairments (3). LS can give rise to a range of complications, including vascular cognitive impairment, depression, and an increased risk of brain hemorrhage or secondary cerebral infarction (4–8). Furthermore, Furthermore, LS frequently follows a clinically silent or only mildly symptomatic course. One study reported that asymptomatic lacunar lesions are often detected incidentally during brain imaging performed for other indications (9). Subtle impairments in both physical and cognitive functions are frequently overlooked, leading to adverse long-term consequences (10). Given these risks, further research into LS is essential to elucidate its pathophysiological mechanisms and to inform prevention and treatment strategies.

Accumulating evidence indicates that LS is strongly associated with endothelial dysfunction and disruption of the blood–brain barrier (11). Additionally, patients with sporadic symptomatic LS may exhibit secondary changes in adjacent white matter, particularly within the subcortical and periventricular regions (12–14). Over time, these changes may result in cavitation, which is linked to poor motor function recovery and accelerated cognitive decline. Together, these findings highlight the need for further research into the vascular and neurodegenerative mechanisms of LS.

LS alters neuronal activity and disrupts complex cortical–subcortical brain networks. For example, magnetic resonance imaging (MRI) studies comparing LS patients with healthy controls (HCs) have revealed that asymptomatic individuals with LS exhibit significant gray matter volume reductions in regions such as the anterior cingulate cortex, caudate nucleus, superior temporal gyrus, and insula (15). Moreover, a study by Lawrence et al. (73) that included 97 symptomatic LS patients demonstrated a marked loss of structural network integrity in these patients, which may underlie their cognitive impairments.

Although much of the existing research into LS has focused on structural and functional alterations, its metabolic characteristics—particularly, metabolic connectivity and network organization—remain largely unexplored. Examining LS from a metabolic perspective is therefore of considerable importance. Positron emission tomography (PET) can circumvent the limitations that are inherent in blood oxygen level–dependent MRI, which relies on neurovascular coupling (16). Because neurotransmission and signal transduction are energy-demanding processes that depend on glucose, ^18^F-fluorodeoxyglucose (^18^F-FDG) has become the most widely used PET tracer for *in vivo* studies of cerebral glucose metabolism (17, 18). Moreover, integrating PET with MRI allows the simultaneous assessment of metabolic and hemodynamic changes (19, 20). This approach deepens our understanding of the underlying pathophysiological processes and supports the longitudinal clinical assessment of various central nervous system disorders. Importantly, ^18^F-FDG PET/MRI has been highlighted as an invaluable tool in stroke research beyond the acute phase because it allows the simultaneous assessment of structural lesions, hemodynamics, and metabolism within a single examination, thereby providing deeper insights into subacute and chronic brain remodeling in stroke patients (21).

In the present study, we used ^18^F-FDG PET/MRI to investigate brain alterations in LS. Specifically, we examined glucose uptake, metabolic connectivity, and network topology. By integrating these complementary perspectives, our work aims to advance our understanding of the metabolic underpinnings of LS and their potential clinical implications.

## 2 Method

### 2.1 Participants

This study protocol was approved by the Medical Ethics Committee of Yueyang Hospital of Integrated Traditional Chinese and Western Medicine, Shanghai University of Traditional Chinese Medicine (Approval No. 2020-188). The study was conducted in accordance with the principles of the Declaration of Helsinki. Written informed consent was obtained from all participants prior to enrollment.

In this retrospective study, we reviewed the ^18^F-FDG PET/MRI database of the Panoramic Medical Imaging Diagnostic Center (Shanghai, China) and identified 140 consecutive individuals who underwent brain PET/MRI between January 2021 and December 2021 for routine clinical evaluation. Among these, 70 individuals fulfilled imaging criteria for LS and 70 met criteria for HCs.

**Figure 1:**
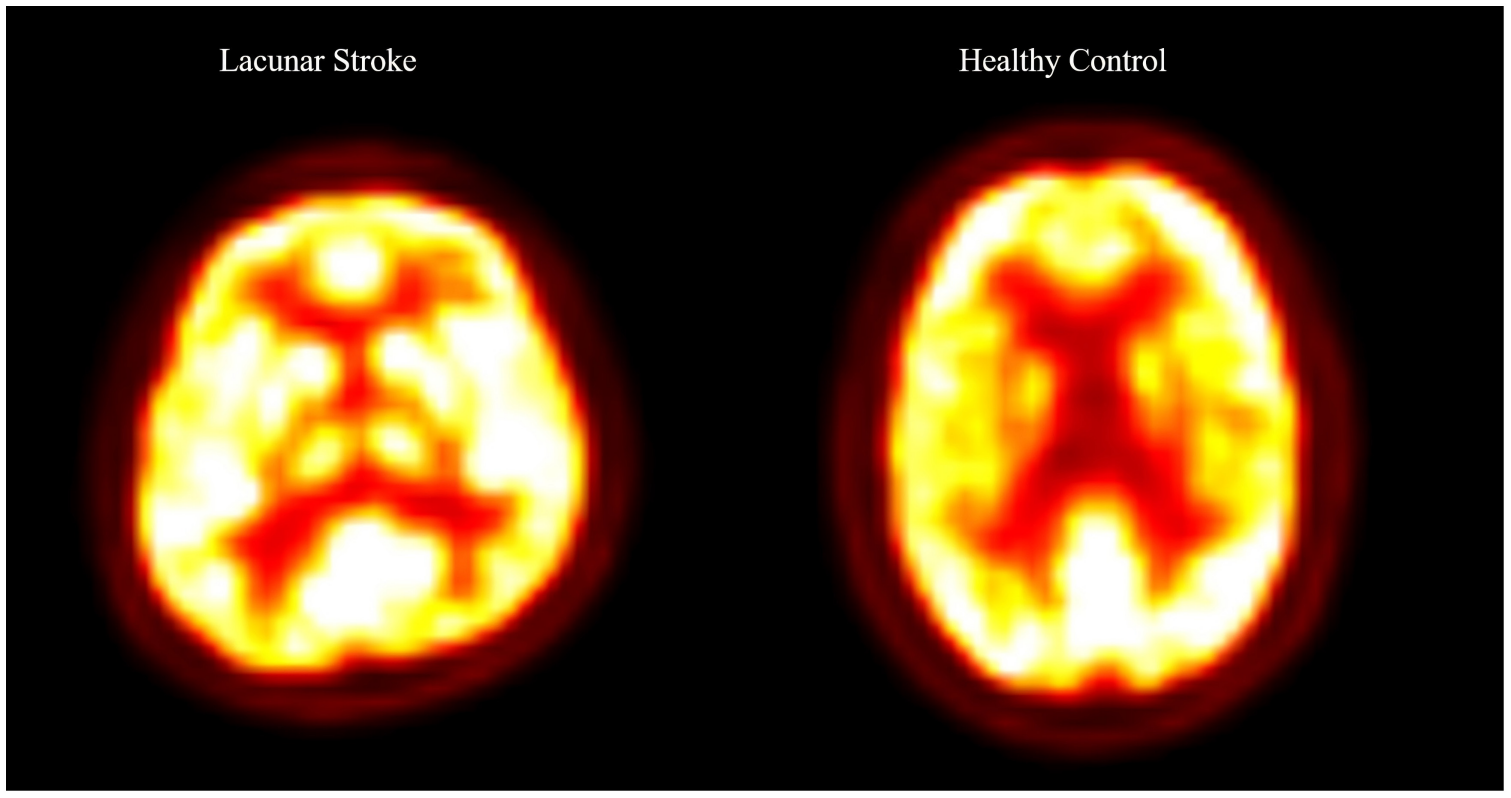
Representative ^18^F-FDG PET/MRI images from a patient with lacunar stroke (LS) and a healthy control (HC).

In the LS group, conventional MRI demonstrated a single chronic lacunar infarct confined to the basal ganglia, located in typical perforator territories (putamen, globus pallidus, caudate head, or internal capsule), with a maximum diameter < 15 mm and no cortical, thalamic, brainstem, or cerebellar involvement. Lacunes were identified on T1-weighted images and confirmed by an experienced neuroradiologist who was blinded to the clinical data. On the basis of clinical records and MRI appearance, lesions were classified as chronic, with an estimated time since the index LS between 6 and 12 months (mean ± SD, 8.3 ± 2.4 months) at the time of PET/MRI. Routine blood tests and liver function tests obtained as part of the standard clinical work-up were available for all participants. Representative PET/MRI images from one LS patient and one HC are shown in Figure 1.

### 2.2 Inclusion and exclusion criteria

The inclusion criteria for LS patients were based on the classification model established in the Trial of Org 10172 in Acute Stroke Treatment (22), as follows: (1) a typical lacunar syndrome without cortical involvement; (2) supporting risk factors such as hypertension and diabetes mellitus; (3) the absence of infarcts that may explain the neurological deficits on computed tomography/MRI, or the presence of subcortical lesions < 15 mm in diameter; and (4) no evidence suggesting a high likelihood of cardioembolism or embolism caused by arterial stenosis >50%. In addition, LS patients were required to be between 18 and 85 years of age, to have a National Institutes of Health Stroke Scale (NIHSS) score < 4 at the time of imaging, and to have at least 6 years of formal education. All LS patients were in the chronic stage of stroke and were scanned between 6 and 12 months after the index LS.

For HCs, the inclusion criteria were: (1) no history of stroke, transient ischemic attack, or other cerebrovascular disease; (2) age between 18 and 85 years; (3) a National Institutes of Health Stroke Scale (NIHSS) score of 0 at the time of imaging; and (4) at least 6 years of formal education.

Exclusion criteria for all participants were as follows: (1) a history of other subtypes of stroke such as cortical infarction, large-artery atherosclerosis, or intracerebral hemorrhage; (2) serious neurological diseases, including Alzheimer's disease, Parkinson's disease, epilepsy, or multiple sclerosis and so on; (3) psychiatric disorders; (4) severe general health problems, such as advanced heart failure, kidney failure, liver cirrhosis, or cancer; (5) known autoimmune or systemic inflammatory diseases or active infection, including encephalitis or other inflammatory central nervous system conditions; (6) contraindications to MRI such as metallic implants; or (7) an allergy to ^18^F-FDG or MRI contrast agents.

### 2.3 Image acquisition

Imaging was conducted using a Biograph mMR scanner (Siemens, Munich, Germany). Participants were instructed to fast for ≥6 h before the scan. This fasting requirement also included pausing any tube feeding, glucose-containing intravenous infusions, and parenteral nutrition. Only plain water was permitted, and no sugar or carbohydrates were allowed during this period (23). Blood glucose levels were measured before injection to ensure that values were < 200 mg/dl (24). Participants' heads were comfortably stabilized with foam padding within the head coil to minimize motion, and list-mode data were acquired throughout the scan to permit data-driven motion correction during reconstruction if necessary. During scanning, participants lay supine with their eyes closed in a resting state. The PET/MRI acquisition covered five bed positions, with a total duration of 40–50 min. The injected dose of ^18^F-FDG was 3.7 MBq/kg. Images were reconstructed in three dimensions with a slice thickness of 2.03 mm, a matrix size of 172 × 172, and an in-plane resolution of 4.17 × 4.17 mm^2^. Post-reconstruction, a Gaussian filter with a full width at half maximum of 4.0 mm was applied. For attenuation correction, MR images were acquired during breath-hold using a dual-echo spoiled gradient-echo Dixon sequence with fat-water separation (TE1 = 1.23 ms, TE2 = 2.46 ms, TR = 3.6 ms, flip angle = 10°). The resulting attenuation maps were generated by segmenting the Dixon images into air, fat and soft-tissue classes.

### 2.4 Data preprocessing

PET data preprocessing was performed using SPM12 software (https://www.fil.ion.ucl.ac.uk/spm/software/spm12/) on the MATLAB 2021a platform (MathWorks, Inc., Natick, MA, USA). Images were first converted from DICOM to NIfTI format. The anterior commissure was set as the reference point for origin correction. The T1-weighted image was spatially normalized to the standard Montreal Neurological Institute space, and the resulting transformation parameters were applied to the co-registered PET images for spatial normalization. To enhance the signal-to-noise ratio, all images were smoothed with a Gaussian kernel of 6 × 6 × 6 mm^3^.

### 2.5 Metabolism, metabolic connectivity, and network construction

PET images were segmented into 90 brain regions using the Automated Anatomical Labeling atlas (AAL atlas), excluding the cerebellum (25, 26). In this study, the standardized uptake value (SUV) for each ROI was operationally defined as the raw uptake value divided by the subject's mean whole-brain uptake (27).

Metabolic connectivity between each ROI and the remaining 89 regions was assessed using Pearson correlation coefficients. Group-level metabolic networks were constructed using the Brain Connectivity Toolbox (version 2017-01-15; https://www.brain-connectivity-toolbox.net/). In these networks, ROIs were modeled as nodes and correlation coefficients were modeled as edges. Networks were thresholded across a sparsity range of 0.10–0.50 (step = 0.01) to generate an undirected, unweighted binary graph (28). Graph theory was then used to analyze the topological characteristics of the brain network.

### 2.6 Network topological metrics

We calculated both global and nodal network properties. Global metrics included characteristic path length, clustering coefficient, global efficiency, and local efficiency, as well as small-world measures. In this framework, the normalized clustering coefficient (γ) and normalized characteristic path length (λ) quantify the relative clustering and path length of the empirical network compared with a random network, whereas the small-worldness index σ = γ/λ provides a composite measure of small-world organization (29, 30). Local properties include degree, betweenness, and node efficiency, which reflect local information transmission efficiency (31).

### 2.7 Statistical analysis

All analyses were performed using SPSS 26.0 (IBM Corp., Armonk, NY, USA). Data distributions were examined for normality. Normally distributed variables were compared using independent-sample *t*-tests and are reported as the mean (standard deviation). Non-normally distributed variables were compared using Mann–Whitney *U* tests and are presented as the median (interquartile range). Categorical variables were analyzed using χ^2^ tests. A two-tailed *p*-value < 0.05 was considered significant.

For metabolic connectivity, edge-wise *p*-values were controlled using false discovery rate (FDR) correction (*p* < 0.05). For network-level graph metrics, group differences were assessed with a non-parametric permutation test (5,000 random relabellings of group membership), and empirical *p*-values were derived from the resulting null distributions.

SUV values from brain regions that exhibited significant between-group differences in glucose metabolism were extracted and correlated with blood and liver function parameters to evaluate the associations between cerebral metabolism and peripheral biomarkers.

## 3 Results

### 3.1 Demographic characteristics

We identified significant differences in age, body mass index (BMI), smoking history, and drinking between the two groups (all *p* < 0.05). No significant differences were observed in hypertension, hyperglycemia, or hyperlipidemia (all *p* > 0.05). Both the LS and HC groups showed a marked male predominance (LS: 85%; HC: 75%). Although the difference in sex distribution between groups did not reach formal statistical significance (*p* = 0.072), the imbalance warrants consideration as a potential confounding factor (Table 1).

**Table 1:** We found significant differences in age, BMI, smoking history, and drinking (all *p* < 0.05).

**Characteristic**	**Lacunar Strok (*n* = 70)**	**Healthy control (*n* = 70)**	**T/Z/χ2**	***P*-value**
Male, *n* (%)	52 (74.3%)	42 (60%)	3.238	0.072
Age, mean ± SD	53.9 ± 1.2	50.3 ± 0.7	2.519	0.013
BMI, median (Q1, Q3)	24.64 (22.71, 24.64)	23.9 (20.97, 25.60)	2.180	0.029
Hypertension, *n* (%)	23 (32.9%)	13 (18.5%)	3.739	0.053
Hyperglycemia, *n* (%)	10 (14.2%)	6 (8.6%)	1.129	0.288
Hyperlipidemia, *n* (%)	14 (20.0%)	20 (28.6%)	1.398	0.237
Smoking, *n* (%)	10 (14.3%)	1 (1.4%)	7.992	0.005
Drinking, *n* (%)	6 (8.6%)	0 (0.0%)	4.453	0.037

No between-group difference was found in other characteristics, including hypertension, hyperglycemia, hyperlipemia (all p > 0.05).

### 3.2 Metabolism

**Figure 2:**
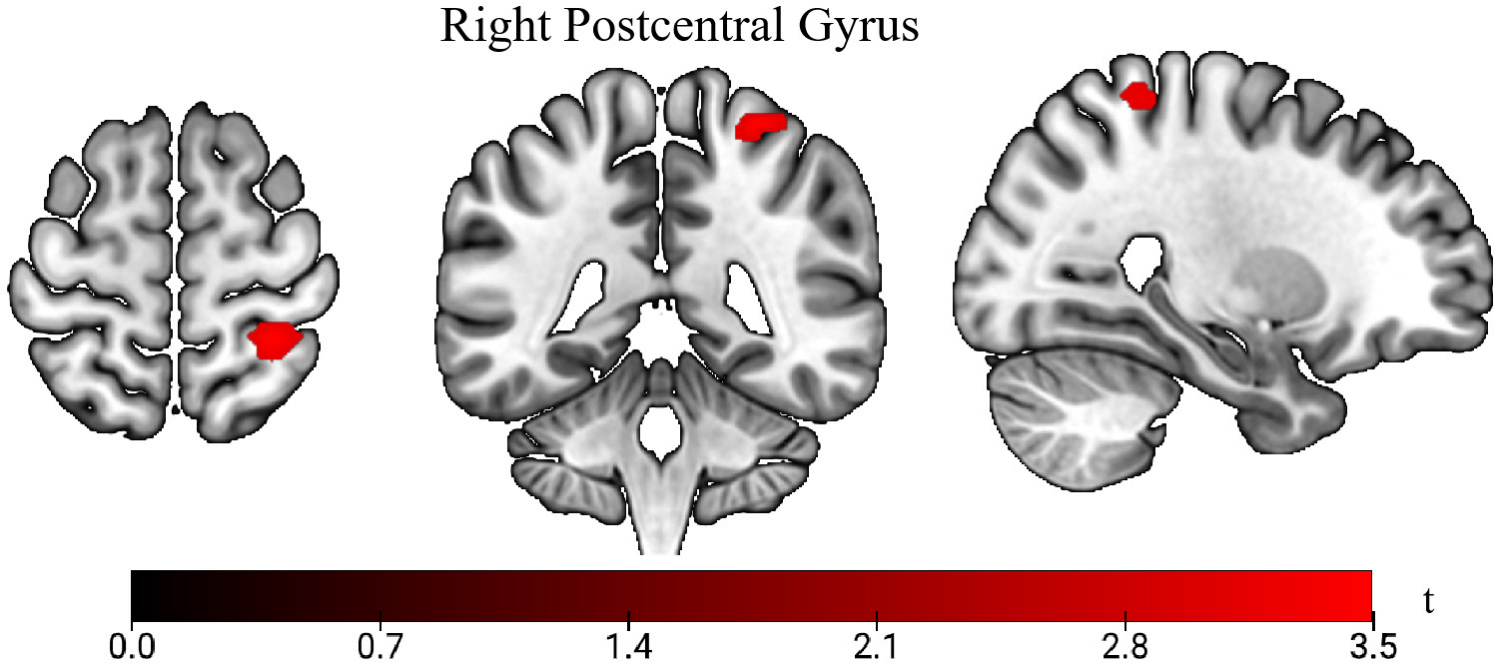
Comparison of standardized uptake values (SUVs) between lacunar stroke (LS) patients and healthy controls (HC). The *t*-value indicates SUV intensity. Areas in red show higher uptake in the LS group vs. the HC group.

We use age, gender, and BMI as covariates for metabolic analysis. Whole-brain voxel-wise comparison of ^18^F-FDG uptake revealed a significant cluster of higher SUVs in the right postcentral gyrus in the LS group compared with the HC group (*p* < 0.001; Figure 2). At the chosen statistical threshold, no regions exhibited significantly lower uptake in the LS group relative to the HC group.

### 3.3 Metabolic connectivity

**Figure 3:**
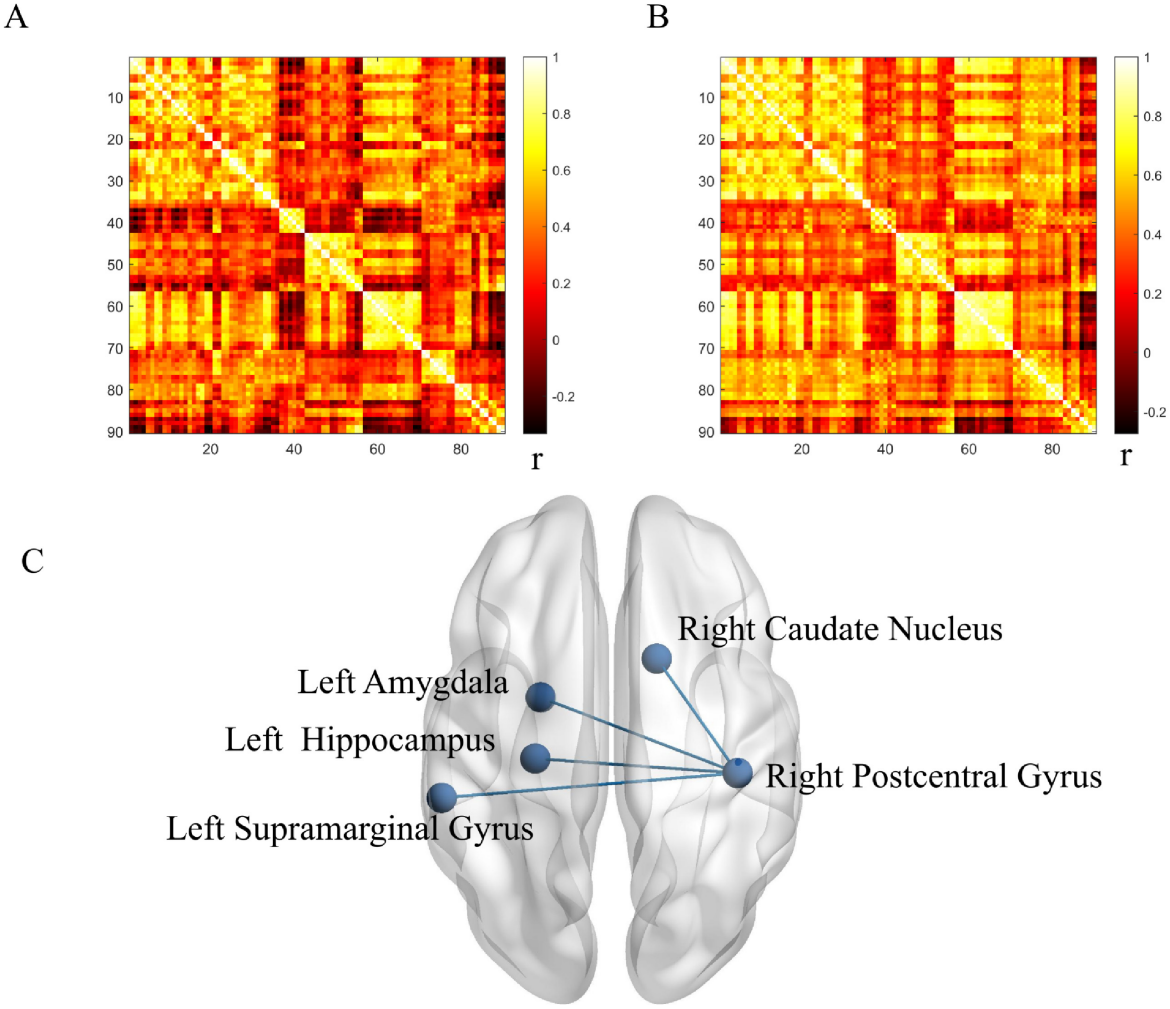
Metabolic connectivity in lacunar stroke (LS) and healthy control (HC) groups. **(A)** Correlation coefficients in the LS group. **(B)** Correlation coefficients in the HC group. **(C)** Between-group comparison. *r* indicates Pearson correlation coefficient. Blue denotes reduced connectivity in the LS group.

Using the right postcentral gyrus as the seed region, metabolic connectivity was calculated between this seed and the remaining 89 AAL atlas regions. Compared with HCs, LS patients showed significantly lower metabolic connectivity between the right postcentral gyrus and the right caudate nucleus, left amygdala, left hippocampus, and left supramarginal gyrus (all *p* < 0.001; Figure 3).

### 3.4 Metabolic network properties

**Figure 4:**
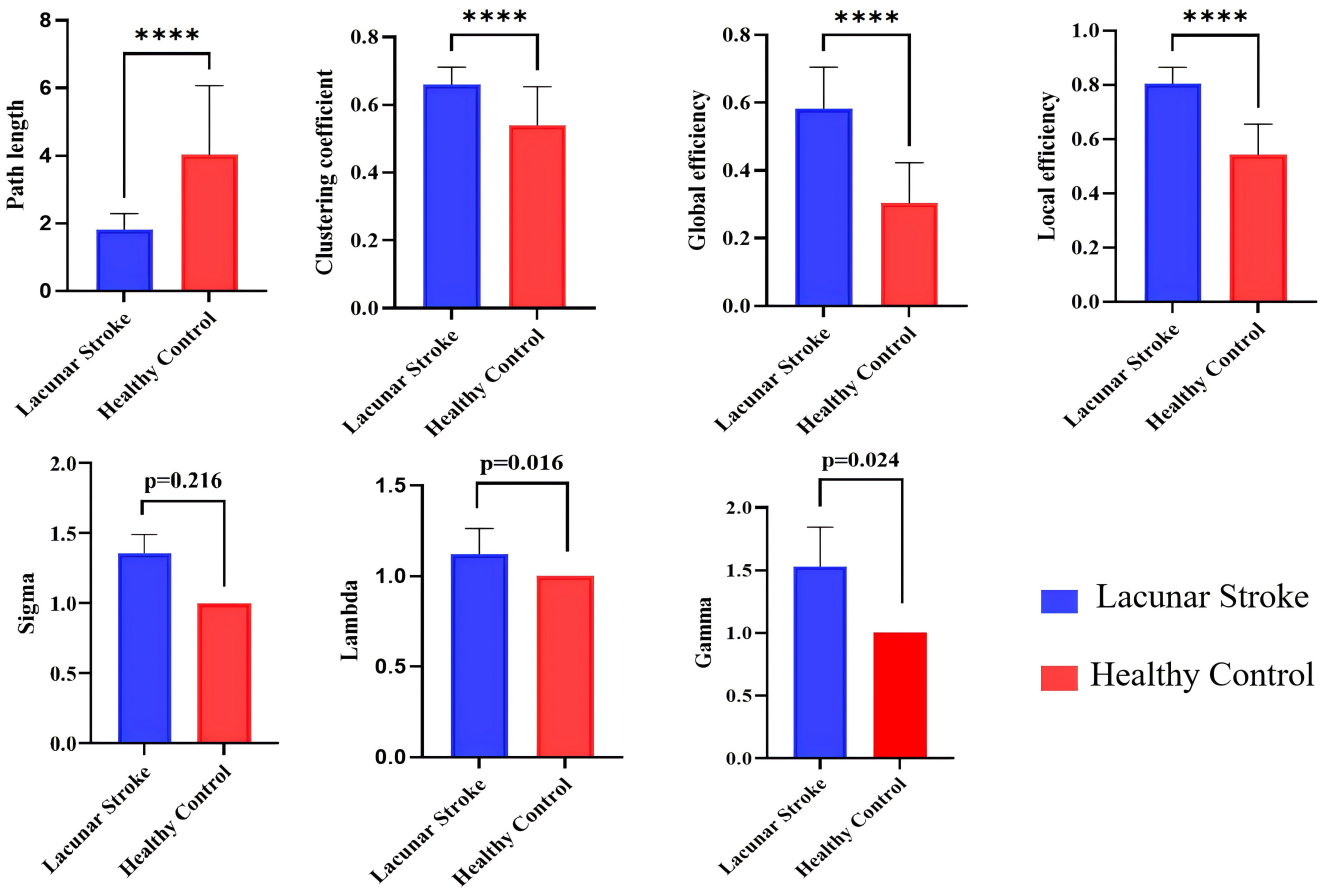
Comparison of global attributes between lacunar stroke (LS) patients and healthy controls (HCs). Blue represents the LS group, while red represents the HC group. ****p < 0.001.

Compared with the HC group, the LS group demonstrated higher clustering coefficient, global efficiency, local efficiency, and shorter path length (all *p* < 0.001). Small-world properties were also altered, with higher γ (*p* = 0.024) and λ (*p* = 0.016) in the LS group but no difference in σ between the two groups (Table 2 and Figure 4).

**Table 2:** The LS group had significantly higher clustering coefficient, global efficiency, and local efficiency than the HC group, while path length was significantly lower (*p* < 0.001).

**Global network properties**	***p*-value**
Path length	<0.001
Clustering coefficient	<0.001
Global efficiency	<0.001
Local efficiency	<0.001
Sigma	0.216
Gamma	0.024
Lambda	0.016

The LS group exhibited an efficient small-world topology, with higher Gamma (*p* = 0.024) and Lambda (*p* = 0.016) compared to the HC group, although there was no significant difference in Sigma between the two groups (Figure 2).

Compared with the HC group, the LS group exhibited a higher degree in the right superior temporal gyrus (*p* = 0.002), greater efficiency in the superior part of the right middle frontal gyrus (*p* = 0.004), and a higher degree in the left insula (*p* = 0.002; Table 3).

**Table 3:** Compared to the HC group, the LS group exhibited an increase in degree in the right superior temporal gyrus (*p* = 0.002), an increase in efficiency in the upper part of the right middle frontal gyrus (*p* = 0.004), and an increase in degree in the left insula (*p* = 0.002).

**Nodal characteristic**	**Brain region**	***p*-value**
Patient > HC Degree	Right_Superior temporal gyrus	0.002
Efficiency	Right_Superior frontal gyrus	0.004
Patient < HC Degree	Left_Insula	0.002

### 3.5 Correlation analysis

**Figure 5:**
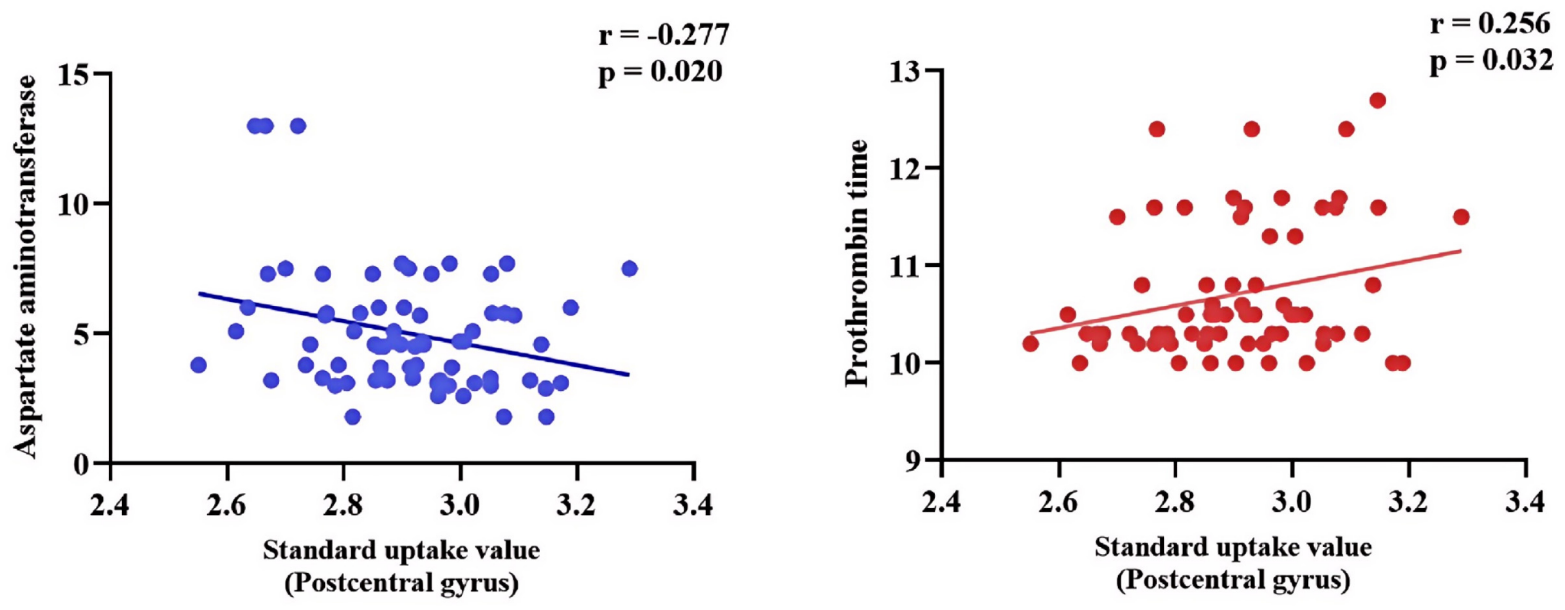
Correlation analysis between SUV and aspartate aminotransferase (AST), as well as between SUV and prothrombin time (PT), in lacunar stroke patients. Blue indicates a negative correlation, while red represents a positive correlation.

In the LS group, SUV in the right postcentral gyrus was significantly negatively correlated with serum aspartate aminotransferase (AST; *r* = −0.277, *p* = 0.020) and significantly positively correlated with prothrombin time (PT; *r* = 0.256, *p* = 0.032; Figure 5). No significant correlations between SUV and these laboratory markers were observed in the HC group.

## 4 Discussion

The application of integrated PET/MRI in LS is mechanistically grounded in the ability of ^18^F-FDG PET to probe cerebral glucose metabolism, thereby providing a direct window into synaptic and neuronal integrity (32). This is particularly relevant given that LS pathogenesis involves endothelial dysfunction and neurovascular unit injury, which can disrupt metabolic (33). While CT and structural MRI excellently delineate the macroscopic infarct, they cannot assess the functional/metabolic sequelae in structurally preserved tissue (34). Although functional MRI adds a hemodynamic dimension, the blood oxygen level-dependent signal is an indirect proxy for neural activity and is itself susceptible to the impaired neurovascular coupling that characterizes cerebral small vessel disease—the primary etiology of LS.

By comparison, ^18^F-FDG PET quantifies regional glucose utilization, offering a more direct index of synaptic integrity and neuronal viability. This capability allows the detection of cortical and subcortical areas of hypometabolism that are not evident as lacunes or white matter hyperintensities on conventional imaging (35). Critically, the ^18^F-FDG-PET signal is coupled to neuronal activity through specific biochemical pathways: the release of excitatory glutamate activates sodium/potassium pumps, stimulating glucose consumption via aerobic glycolysis (36). This energy expenditure serves as a proxy for directional signaling, as increased local metabolism indicates heightened afferent neuronal activity (37). In support of this, our prior work in subjects with white matter hyperintensities demonstrated that an individualized metabolic contribution index derived from ^18^F-FDG PET/MRI sensitively captured network reorganization and distinguished patients from controls, underscoring the potential clinical relevance of metabolic network analysis in small vessel disease (26). As a contribution to our understanding of LS, the present study was the first to use ^18^F-FDG-PET/MRI to investigate brain metabolism in LS patients through glucose uptake, metabolic connectivity, and metabolic networks. We examined brain metabolism at the group level to reveal differences between LS patients and HCs, thus providing clues for subsequent in-depth mechanistic studies.

The SUV is the ratio of activity concentration in the target tissue or lesion to the total body activity concentration, thereby reflecting glucose metabolism (38). The postcentral gyrus is involved in sensory information processing (39), and sensory deficits, including hypoesthesia and dysesthesia, are a major sequela of LS (40). Patients with pure sensory and sensorimotor strokes, as well as those with ataxic hemiparesis accompanied by sensory impairment, experience sensory dysfunction. Additionally, the postcentral gyrus is involved in integrating proprioceptive information with motor information (41). These findings align with our results of increased glucose metabolism in the postcentral gyrus, which may reflect compensatory mechanisms related to sensory and sensorimotor integration deficits in patients. Heightened metabolic activity in the postcentral gyrus may indicate an increased effort to process sensory inputs or adapt to impaired integration with motor functions.

In addition, a potential confounding factor in the interpretation of regional hypermetabolism is the contribution of persistent neuroinflammation. Activated glial cells, particularly microglia and astrocytes, are known to undergo metabolic reprogramming and increase their glucose consumption in response to brain injury (42). As these cells are key drivers of neuroinflammation, their activation can lead to increased FDG uptake, a phenomenon documented in various neurological conditions (43). Therefore, the focal hypermetabolism observed in our LS patients may represent not only compensatory neuronal and synaptic hyperactivity but also concurrent neuroinflammatory processes.

We used the postcentral gyrus as a seed region and conducted a metabolic connectivity analysis with the remaining 89 ROIs. We observed reduced metabolic connectivity in the LS group between the right postcentral gyrus and the right caudate nucleus, left amygdala, left hippocampus, and left supramarginal gyrus. The caudate nucleus plays a key role in motor execution and is also crucial for learning, memory, reward, motivation, and emotion (44, 45). The hippocampus is central to human memory research; it is crucial for learning and memory and is closely related to the regulation of emotions (46). The amygdala is essential for behaviors related to emotions and motivation, and is associated with advanced cognitive functions (47). The supramarginal gyrus is involved in auditory–motor integration and plays a role in language-related short-term memory processes (48, 49). Previous studies have noted deficits in executive function, learning, and memory in LS patients (50), consistent with our findings. Apathy is also reported to be a sequela of small vessel disease (51); this aligns with our discovery of abnormalities in emotion-related brain regions in LS patients.

At the microvascular level, endothelial dysfunction and neurovascular unit injury in LS may impair the normal matching between neural activity, blood flow and glucose delivery (11), thereby contributing to spatially heterogeneous metabolic connectivity and selectively vulnerable cortico–limbic circuits. In addition to local effects of basal ganglia lacunes, these long-range disconnections may also reflect network-level diaschisis, potentially including transcallosal effects on structurally intact association and limbic regions. Such diaschisis-related disruption of large-scale coupling provides a plausible mechanism linking focal lacunar damage to widespread disturbances in cognition and emotion. The observed reduced metabolic connectivity suggests that LS may involve not only local brain damage but also broader dysfunction in brain networks, potentially affecting higher-order cognitive functions such as memory and emotion.

From a topological perspective, we further examined the brains of LS patients and HCs. Our findings indicate that the network exhibits improved overall information transmission efficiency—as evidenced by higher clustering coefficients, enhanced global and local efficiency, and reduced path length—in LS patients. Small-world networks are considered to represent optimized network organization for segregated and integrated information processing (52). Increased gamma and lambda suggest a more efficient network. This may represent a compensatory mechanism that is triggered by endothelial cell damage and disruption of the blood–brain barrier, both of which are consequences of small vessel disease and contribute to vascular inflammation (33). Additionally, the observed increase in global properties might indicate compensatory and adaptive processes that are aimed at optimizing and preserving glucose metabolism within the neural network of the brain.

All LS patients in the present study were examined 6–12 months after the LS. At this chronic stage, the most intense acute innate inflammatory response and peripheral immune-cell infiltration have largely subsided, whereas longer-term processes such as diaschisis resolution, neural plasticity and network reorganization predominate (53, 54). One previous study has reported that small-world properties tend to be reduced in the subacute phase after stroke but increase again during the chronic stage, in parallel with ongoing network reorganization and plasticity (55). In HCs, network connectivity was relatively simple and homogeneous across individuals, consistent with a streamlined and efficiently organized architecture. In stroke patients, by contrast, the more complex connectivity pattern may reflect the emergence of additional or strengthened connections that bypass damaged pathways, providing evidence for compensatory reorganization within the residual network (56). Brain networks typically exhibit a small-world topology, characterized by dense local clustering together with a relatively small number of long-range connections that maintain short path lengths between any two regions. Such small-world architecture supports both globally and locally efficient parallel information transfer, while achieving an economical balance between high communication efficiency and limited wiring cost (57). The increased small-world indices observed in LS may indicate a more metabolically costly network configuration, in which enhanced clustering and connectivity require greater energy expenditure to maintain (58). Part of the observed hypermetabolism and network reconfiguration may reflect maladaptive plasticity, such as inefficient recruitment of auxiliary regions or circuits that increases metabolic cost without necessarily improving behavioral performance.

Changes in local network properties were observed in the right superior temporal gyrus, right superior frontal gyrus, and left insula. The superior temporal gyrus is involved in auditory processing and social cognition, and participates in emotion and higher-order cognitive functions (59, 60). The superior frontal gyrus is part of the dorsolateral prefrontal cortex (61), which is involved in planning complex cognitive behaviors and the expression of personality (62). The insula is involved in interoception, pain, and cognition (63). Our findings of changes in local network properties in areas related to emotion and cognition are therefore consistent with our metabolic connectivity results. A previous MRI study of stroke patients with mild cognitive impairment has reported alterations in brain regions such as the precuneus, insula, frontal lobe, hippocampus, and temporal lobe (64). Some of these changes overlap with the regions of abnormal functional connectivity that were identified in our study.

Our results showed that age, BMI, smoking, and alcohol consumption differed significantly between LS patients and HCs. With increasing age, the risk of developing LS becomes higher. BMI, smoking, and alcohol consumption are associated with unhealthy lifestyles and lipid metabolism abnormalities, which is consistent with previous studies (65). Both the LS and HC groups showed a clear predominance of male participants. In addition, the predominance of male participants likely reflects the underlying epidemiology of lacunar and ischemic stroke and the characteristics of patients presenting to our center, as several population-based studies have reported a higher incidence of ischemic and LS in men than in women, particularly among middle-aged individuals with vascular risk factors such as hypertension, smoking and dyslipidemia (66). This male predominance should be taken into account when interpreting our findings, as it may limit their generalizability to female patients; future studies with more balanced sex distributions or sex-stratified analyses are therefore warranted. However, in contrast to other studies, we did not find significant differences between the two groups regarding hypertension and diabetes (67). This finding might be attributed to the relatively small sample size in our study, or it may be influenced by age. The average age of participants in our cohort was < 55 years, which may not be sufficient for the development of these conditions to a significant extent (68).

We identified correlations between the SUV in the postcentral gyrus of the LS group and routine blood test results and liver function indicators. The SUV was positively correlated with PT and negatively correlated with AST. PT reflects the time that is required for blood clot formation and is used to assess coagulation efficiency (69). Previous research indicates that PT is associated with stroke, especially hemorrhagic stroke (70). The positive correlation between SUV and PT suggests that higher metabolism may increase coagulation-related risks. AST (also known as glutamate oxaloacetate transaminase) is the only enzyme that has been demonstrated to be directly and independently associated with infarct size in stroke (71). Notably, higher AST levels are reported to predict better stroke outcomes because AST helps to clear excitotoxic extracellular glutamate (72). Our results indicate that higher local metabolism is correlated with lower AST levels. An elevated metabolism may be associated with worse patient outcomes because reduced AST levels may impair the clearance of glutamate, thereby affecting neuronal survival and functional recovery.

In conclusion, we identified significant differences in metabolism, metabolic connectivity, and metabolic networks between LS patients and HCs. These changes were primarily observed in brain regions and networks that are associated with sensory processing, motor execution, memory, and higher cognitive functions. Our findings provide further insights into the brain alterations that occur in LS, thus offering new perspectives for understanding and treating LS. In addition, these PET/MRI-derived metabolic and connectivity alterations may provide new insights into therapeutic targeting and help to identify candidate brain networks for future intervention strategies in LS.

There are several limitations to our study. First, our sample size was limited and may not fully represent the broader LS population. Second, we did not stratify the participants by age groups, which presents challenges for interpreting the results and drawing precise conclusions. Third, our study population was predominantly male. This sex imbalance likely reflects the epidemiological characteristics of LS and the patients presenting to our hospital during the recruitment period, among whom men with vascular risk factors are overrepresented. Nevertheless, this limitation may limit the generalizability of our findings to female patients, and future studies with more balanced sex distributions are warranted. Future prospective studies with standardized lesion characterization are needed to clarify how lesion burden and lesion age influence the observed metabolic and network alterations. A further limitation is that we did not apply explicit partial-volume effect correction, which may lead to underestimation of FDG uptake in small deep gray-matter structures such as the thalamus, caudate and hippocampus. In addition, the Dixon-based attenuation correction used on our PET/MR system does not explicitly model bone, potentially causing a slight underestimation of activity near the skull base; however, these systematic biases are present in both the LS and HC groups and are therefore unlikely to alter the primary comparative findings. Lastly, the present study lacked a follow-up period. Considering the long-term neurological changes that are associated with LS, follow-up studies are necessary for a more comprehensive understanding. Future research should therefore focus on increasing the sample size, dividing LS patients into age groups, and incorporating a follow-up period to better investigate the metabolic mechanisms of the brain in LS.

## Data Availability

The raw data supporting the conclusions of this article will be made available by the authors, without undue reservation.
